# Proteomic Analysis and Expression of Selected Genes During the Early Somatic Embryogenesis of *Jatropha curcas* L.

**DOI:** 10.3390/ijms26136384

**Published:** 2025-07-02

**Authors:** Anamarel Edzná Medina-Hernández, Ileana Vera-Reyes, Emmanuel Ríos-Castro, Juan José Torres-Ruiz, Teresa Ponce-Noyola, Gabriela Trejo-Tapia, Adriana Garay-Arroyo, Josefina Barrera-Cortés, Ana C. Ramos-Valdivia

**Affiliations:** 1 Departamento de Biotecnología y Bioingeniería, Centro de Investigación y de Estudios Avanzados del Instituto Politécnico Nacional (Cinvestav), Ciudad de Mexico 07360, Mexico; aehernandezm@cinvestav.mx (A.E.M.-H.); juanj.torres@cinvestav.mx (J.J.T.-R.); tponce@cinvestav.mx (T.P.-N.); jbarrera@cinvestav.mx (J.B.-C.); 2 Centro de Investigación en Química Aplicada, Departamento de Biociencias Y Agrotecnología, Saltillo 25294, Coahuila, Mexico; ileana.vera@ciqa.edu.mx; 3 Unidad de Genómica, Proteómica y Metabolómica, LaNSE, Centro de Investigación y de Estudios Avanzados del Instituto Politécnico Nacional (Cinvestav), Ciudad de Mexico 07360, Mexico; eriosc@cinvestav.mx; 4 Departamento de Biotecnología, Centro de Desarrollo de Productos Bióticos, Instituto Politécnico Nacional, Yautepec 62730, Morelos, Mexico; gttapia@ipn.mx; 5 Departamento de Ecología Funcional, Instituto de Ecología, Universidad Nacional Autónoma de México, Ciudad de Mexico 04510, Mexico; agaray@iecologia.unam.mx

**Keywords:** *Jatropha curcas*, early somatic embryogenesis, embryo liquid cultures, proteomic analysis, gene expression, *BBM*, *AGL15*, *SERK*, *IAA26*, *eIF3f*

## Abstract

*Jatropha curcas* L. is a shrub of the Euphorbiaceae family with non-toxic varieties found in Mexico that holds significant potential for biofuel production and other industrial applications. However, its limited in vitro regenerative capacity is a barrier to the development of productive species. Somatic embryogenesis (SE) offers a strategy to establish a regeneration system to overcome these challenges and enable genetic improvement. In this work, proteomic and gene expression analyses were utilized to identify key factors involved in SE induction in a non-toxic variety of *J. curcas*. Two-dimensional electrophoresis (2-DE) in combination with mass spectrometry was used to compare the proteomes of pre-globular and globular somatic embryos. RT-qPCR was used for gene expression analysis of the *BBM*, *AGL15*, *SERK*, *IAA26* and *eIF3f* genes. The globular stage showed enrichment in the pathways related to carbohydrate and energy metabolism, protein folding, and stress response. In addition, the gene expression analysis of selected genes revealed a significantly elevated expression of *BBM*, *AGL15*, and *IAA26* in globular embryos compared to pre-globular embryos. In contrast, *SERK* expression was low, and *eIF3f* expression remained unchanged between stages. These expression patterns may contribute to developmental arrest at the globular stage. These findings provide new insights into the molecular mechanisms regulating early SE in *J. curcas* and offer potential strategies for improving its propagation and industrial applications.

## 1. Introduction

*Jatropha curcas* L. is a shrub from the Euphorbiaceae family. It is economically significant for biofuel production due to its oil-rich seeds, with potential additional applications in pharmacology [1,2].

Two main types of *J. curcas* are recognized based on the presence or absence of phorbol esters of heat-tolerant, toxic diterpenes. These compounds are found primarily in toxic varieties, while non-toxic varieties lack them or contain significantly lower amounts of these diterpenes [3].

Non-toxic varieties found in Mexico are particularly valuable for both animal and human consumption as they do not pose a risk of tumor-promoting activity [3]. Li et al. [4] proposed the Central Depression of Chiapas, Mexico, as the species’ center of origin, while Vandepitte et al. and Zavala-del Angel et al. [5,6] identified a broader region of origin and diversification in northern Veracruz, where non-toxic varieties are prevalent.

The industrial use of *J. curcas* faces several limitations, including susceptibility to diseases, asynchronous fruit ripening, and high variability in yield per plant, which complicates the development of productive varieties [7]. Consequently, *J. curcas* remains in the early stages of domestication and requires significant genetic improvement for commercial use. This highlights the importance of applying biotechnological tools in its development [8].

Somatic embryogenesis (SE) is a process that involves the formation of embryos genetically identical to the mother plant without gametic fusion. This process relies on the reprogramming of somatic cells into totipotent cells capable of regeneration and it is used as a platform for genetic transformation in agronomically important species [9,10]. It is particularly useful for studying plant embryogenesis, as it provides a dais from which to investigate the molecular and physiological factors that determine cellular plasticity and embryogenic transition [11,12].

In dicotyledonous species, SE begins with an early stage that includes the acquisition of embryogenic competence, the establishment of embryogenic identity, cellular proliferation, and the formation of pro-embryos, followed by the development of globular embryos [13,14,15]. Embryogenic competence refers to the capacity of somatic cells to perceive and respond to endogenous or exogenous signals, which trigger the transition into an embryogenic identity [13]. The subsequent SE stages involve further embryo development through the heart, torpedo, and cotyledonary stages, leading to the formation of viable plants [14]. In indirect SE, an intermediate callus phase occurs, during which embryogenic competence is acquired following callus formation [14].

Although *J*. *curcas* is considered a recalcitrant species due to its limited responsiveness to tissue culture, there are reports of success in in vitro clonal propagation, as well as the establishment of cell suspension cultures for metabolite production [16]. Additionally, SE protocols have been developed, focusing on optimizing the media and conditions for inducing SE.

However, these protocols are still limited in scale compared to those of other species that have achieved desirable genotypes for industrial applications [5,6,8]. Despite its biotechnological and industrial potential, research into the molecular mechanisms underlying the SE in *J. curcas* remains scarce. Therefore, integrating omics technologies is essential to better understand plant embryogenesis and overcome current industrial limitations.

Proteomics is a powerful approach for determining the identity, abundance and post-translational modifications of proteins involved in embryonic development. Among the available proteomic methods, two-dimensional gel electrophoresis (2-DE) remains widely used as a separation technique due to its robustness, ability to resolve protein isoforms, and compatibility with other analytical tools. Despite its limitations, such as the co-migration of multiple proteins within a single spot, 2-DE continues to offer valuable insights, especially when combined with mass spectrometry [17]. In this context, the exponentially modified protein abundance index (emPAI) has proven useful for determining the relative contribution of individual proteins to overall spot intensity in comparative proteomic analyses under different biological conditions [18]. In *J. curcas*, proteomics studies have been carried out in various tissues including mature seeds, leaves, roots and endosperms primarily to explore lipid metabolism and seed development [19,20]. However, proteomic studies related to SE in *J. curcas* remain limited, representing a gap in our understanding of the molecular mechanisms governing early embryo development in this species.

Recent studies suggest that transcriptional and chromatin mechanisms involved in cellular reprogramming during SE induction follow a hierarchical organization [9,21]. The first level of this hierarchy involves the developmental stage of the explant, which influences chromatin composition and accessibility. The second level refers to the role of auxins in modulating chromatin dynamics [9,21]. At this level, the AUXIN/INDOLE-3-ACETIC ACID (Aux/IAAs) proteins play a central role by interacting with AUXIN RESPONSE FACTORs (ARFs), thereby regulating the expression of auxin-responsive genes and influencing cell differentiation and reprogramming during SE induction. The third level includes key transcription factors (TFs) such as LEAFY COTYLEDON 1 (LEC1), LEAFY COTYLEDON2 (LEC2), FUSCA 3 (FUS3), BABY BOOM (BBM), WUSCHEL (WUS) and AGAMOUS-LIKE 15 (AGL15), which are known to initiate and enhance SE in *Arabidopsis thaliana* and other model plant species [9,22]. Additionally, translational regulation plays a significant role in SE, involving components such as elongation factors, eukaryotic initiation factors (eIFs), and ribosomal proteins [14,23]. Another important regulator is the SOMATIC EMBRYOGENESIS RECEPTOR KINASE (SERK), which has been associated with SE induction and embryo development in *A. thaliana* and other plant species [24]. SERK has been linked to auxin signaling and plays a role in the acquisition of pluripotency [25]. While most of these molecular mechanisms are characterized in model species, their relevance to *J. curcas* has not yet been established.

To enhance our understanding of the molecular changes underlying the early stages of somatic embryo formation in *J. curcas*, a proteomic analysis was performed to identify differentially abundant proteins involved in the transition from the pre-globular to the globular stage of SE. This analysis utilized 2-D gel electrophoresis coupled with mass spectrometry for protein identification. Additionally, gene expression analysis was performed for selected candidates identified through proteomics, including subunit F of the eukaryotic initiation factor 3 protein (*eIF3-F*) and *IAA26*. Key embryogenic markers such as *BBM*, *AGL15*, and *SERK* were also evaluated. Collectively, these findings provide valuable insights into SE regulation in *J. curcas* and offer promising avenues for improving its propagation and industrial use.

## 2. Results and Discussion

### 2.1. Obtaining Two Developmental Stages of J. curcas Somatic Embryos in Liquid Medium

An indirect SE system for *J. curcas* in a liquid medium was established, and a step-by-step protocol is presented in Supplementary Figure S1. Briefly, hypocotyl explants derived from seedlings of a non-toxic Mexican variety of *J*. *curcas* were initially cultured on callus-induction medium (CIM) supplemented with the synthetic auxin 2,4-dichlorophenoxyacetic acid (2,4-D). The induced callus lost its friability after the first subculture, which necessitated a transfer to callus-maintenance medium (CMM) containing 1.0 mg/L of another auxin 1-naphthaleneacetic acid (NAA) and 0.01 mg/L of a cytokinin 6-benzylaminopurine (BAP). This combination of growth regulators has previously been reported to promote friable callus formation from *J. curcas* leaf explants [26].

After two months in CMM, a green friable callus was obtained and subsequently transferred to embryo induction liquid medium (SEIM), which contains a 1/20 concentration of NAA (0.05 mg/L) and a six-fold increase in BAP (0.06 mg/L). Weekly subculturing in SEIM was performed over a period of one month.

**Figure 1:**
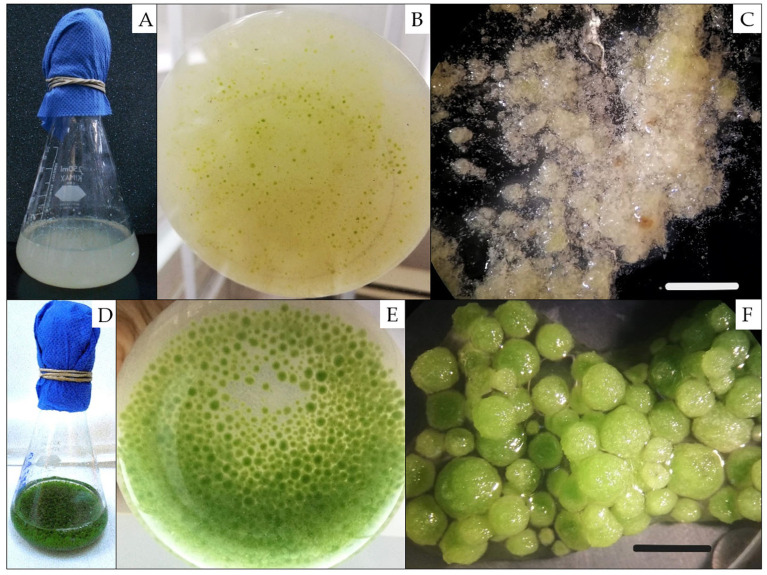
Pre-globular and globular somatic embryos in liquid cultures obtained from non-toxic *J. curcas*. (**A**–**C**) Creamy yellow pre-globular SE observed in (**A**) flask, (**B**) bottom of the flask, (**C**) with a stereoscopic microscope at the third subculture in medium SEIM; (**D**–**F**) green globular SE observed in (**D**) flask, (**E**) bottom of the flask, (**F**) with a stereoscopic microscope. Bar (**C**) = 2.5 mm; bar (**F**) = 5 mm.

Creamy yellow structures resembling embryos with loose cell boundaries emerged during the second subculture and proliferated during the third subculture (Figure 1A–C). These structures were classified as pre-globular somatic embryos. Continued weekly medium replacement, for one month after the third subculture, promoted the development of globular somatic embryos. These structures were spherical, green, homogeneous, and exhibited clearly defined boundaries (Figure 1D–F). Globular embryos, measuring approximately 0.2 to 0.5 cm in size, averaged 84 ± 6 somatic embryos per gram (n = 2) (Figure 1F). However, further development of *J. curcas* SE beyond the globular stage was not achieved despite maintaining cultures in the same induction medium or modifying the conditions by supplementing them with alternative growth regulators, either in liquid or solid media. These observations indicate that additional molecular or physiological cues may be required to support progression to more advanced embryogenic stages.

The induction of somatic embryos in *J. curcas* (Figure 1) could be attributed to the reduction in NAA concentration in the culture medium, a response that has been similarly observed in other *J. curcas* varieties [27] as well as in different plant species [28]. Interestingly, in species such as *Coffea canephora* and *A. thaliana, * a decrease in exogenous auxin levels during SE stimulates the biosynthesis of endogenous indole-3-acetic acid (IAA). This increase in endogenous IAA supports key developmental processes, including the promotion of cell differentiation and root elongation [23,29].

### 2.2. Two-Dimensional Proteome Analysis of Pre-Globular and Globular Somatic Embryos

**Figure 2:**
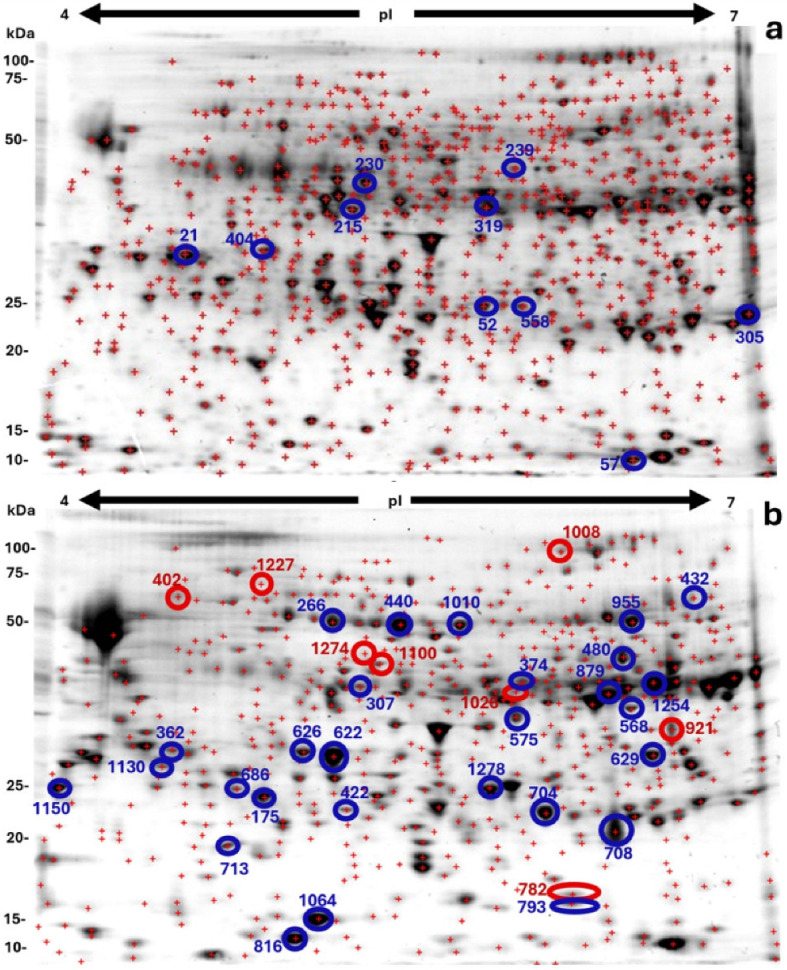
Representative 2D IEF-SDS-PAGE protein profiles of *J. curcas* somatic embryos. The analysis was carried out using ImageMaster^TM^ 2D Platinum software version 7. Molecular masses (kDa) are indicated on the left, and isoelectric points (pI 4–7) are provided above the gel image. (**a**) Protein profile of pre-globular somatic embryos with an average of 654 ± 41 detected spots, and (**b**) protein profile of globular-stage somatic embryos with 552 ± 30 detected spots as indicated by a red cross. The blue and red circles on the gel indicate the protein spots identified through mass spectrometry; the data obtained are presented in Supplementary Tables S1 and S2. Red circles correspond to unique protein spots detected exclusively at the globular stage, while blue circles indicate protein spots that were at least 1.5-fold more abundant in the embryogenic stage than in the other stages. Numbers correspond to spots identified with ImageMaster^TM^ 2D Platinum version 7. First-dimensional isoelectric focusing was performed using 11 cm IPG strips loaded with 200 µg of total protein. Second-dimension separation was carried out using 12.5% SDS-PAGE polyacrylamide gels (CRITERION Cell, Bio-Rad Laboratories, Hercules, CA, USA). Gels were stained with Sypro Ruby (Bio-Rad Laboratories, Hercules, CA, USA).

The proteomic profiles of *J*. *curcas* somatic embryos at two early developmental stages were compared. The 2-DE analysis resulted in a resolution of 654 ± 41 protein spots for the pre-globular stage and 552 ± 30 protein spots for the globular stage. Representative gels of each stage are shown in Figure 2. ANOVA analysis of the gels revealed statistically significant differences in 108 protein spots (*p* ≤ 0.02). Of these, 35 protein spots increased abundance in the globular stage compared to the pre-globular stage, 17 decreased, while 37 protein spots were unique to the pre-globular stage, and 19 were unique at the globular stage.

A total of 50 protein spots were selected for identification by mass spectrometry based on differential abundance or stage-specific presence. This includes 7 spots unique to the globular stage and 43 showed at least a 1.5-fold change in relative spot volume. Spot volume (% volume) was calculated as the ratio of an individual spot’s volume to the total volume of all spots in the gel. Supplementary Material Tables S1 and S2 provide detailed information on each protein spot.

Mass spectrometry identified 152 protein sequences from 46 of the selected spots, whereas four spots did not yield detectable protein sequences. Approximately one-third of the sequences were annotated using the UniProt database version 2015_6, while the remaining sequences were identified using the NCBI BLAST version 2.8.0+ (Supplementary Tables S1 and S2).

In 32 spots where multiple proteins were detected, the emPAI tool [18] was used, either alone or in combination with peptide coverage values greater than 95%, to estimate the most abundant protein (Supplementary Material Tables S1 and S2). Based on the above criteria, 56 proteins were selected for further functional analysis (Table 1 and Table 2).

### 2.3. Functional Classification of Proteins in Pre-Globular and Globular Somatic Embryos

Gene Ontology (GO) annotations for biological processes were successfully retrieved for 39 distinct proteins that were differentially expressed between developmental stages of *J. curcas* somatic embryos (Table 1 and Table 2).

GO slim category enrichment analyses of the proteins in pre-globular compared to globular embryos (Table 1) distinguished a predominant category associated with stress and chemical response processes.

This suggests that early somatic embryo development in *J. curcas* may be closely linked to cellular mechanisms that manage environmental cues.

GO slim category enrichment analyses of the selected proteins in globular embryos compared to pre-globular embryos (Table 2) reveal five major biological process categories: generation of precursor metabolites and energy (23%), protein metabolic processes (12%), photosynthesis (12%), response to stress (8%), and response to chemical stimulus (8%). These enriched categories highlight the metabolomic reprogramming and stress-related responses that accompany the transition from the pre-globular to the globular stage during SE in *J. curcas*.

The activation of stress-related proteins plays a crucial role in regulating the balance between cell proliferation and differentiation, an essential process in SE development [30]. In the pre-globular and globular stages of *J. curcas* somatic embryos, 11 proteins were associated with the GO terms related to stress and chemical responses. In pre-globular somatic embryos, proteins such as glutathione S-transferase PARB (spot 305), superoxide dismutase [Cu-Zn] (spot 57), and lysM domain receptor-like kinase 3 (spot 239) were upregulated by more than 3-fold compared to their levels in globular embryos (Table 1). In contrast, proteins such as glutathione peroxidase (spot 713.1), major allergen Pru ar 1-like proteins (spots 816 and 1064), and cysteine synthase (spots 575 and 1130.1) exhibited higher abundance in globular embryos (Table 2). These findings are consistent with previous reports in *Gossypium hirsutum* L., where stress-related proteins were significantly expressed in globular somatic embryos compared to primary embryogenic callus [31].

Additionally, proteins such as 14-3-3 protein 6 and endo-1,3;1,4-beta-D-glucanase were upregulated in pre-globular embryos, although they were not categorized under stress-related GO terms in *J. curcas*. However, these proteins have been associated with stress responses in other systems. For instance, 14-3-3 proteins were reported in early SE of *Coffea canephora* [32], while the beta-1,3-glucanases proteins have been identified in the callus and pro-embryogenic callus stages during SE in *Elaeis guineensis* Jacq. SE [33].

**Figure 3:**
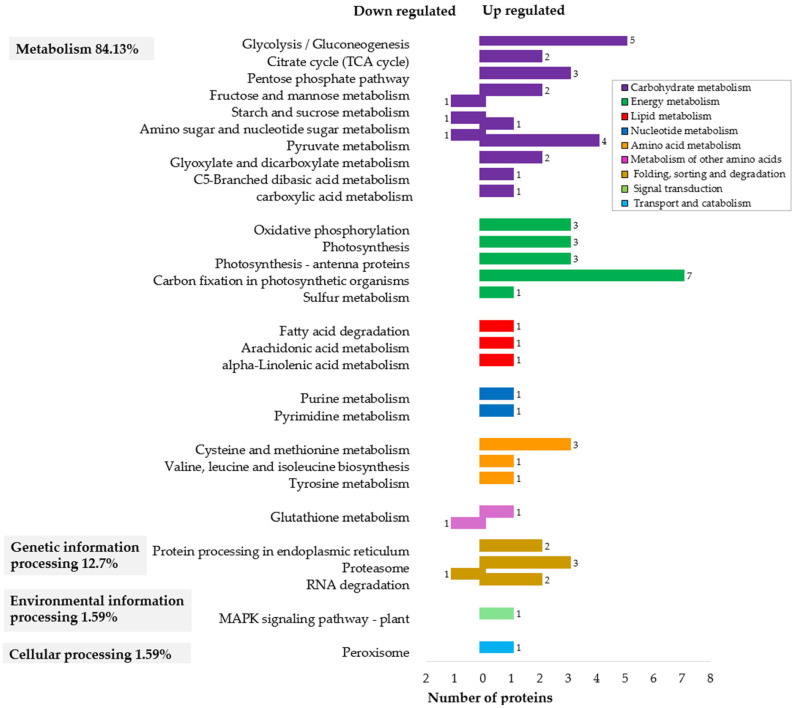
KEGG pathway annotation of 63 proteins from *J. curcas* somatic embryos that were upregulated and downregulated in globular versus pre-globular stages. The analysis revealed four main categories: metabolism, genetic information processing, environmental information processing and cellular processing. Significant enrichment was found in metabolic subcategories, particularly carbohydrate metabolism (24 annotations) and energy metabolism (17 annotations). Distinct colors represent the various subcategories of signaling pathways, and the numbers along the horizontal axis indicate the number of proteins enriched in each pathway. No specific protein markers for somatic embryogenesis (SE) were identified. Data were obtained from the KEGG database version 5 https://www.genome.jp/kegg/pathway.html (accessed on 8 August 2021).

To further explore functional implications, the annotated protein sequences were mapped to the metabolic pathways in the KEGG GENES database version 5 for *J. curcas* https://www.genome.jp/kegg/pathway.html (accessed on 8 August 2021). Four major pathway categories were identified: metabolism (84.13%), genetic information processing (12.7%), environmental information processing (1.59%), and cellular processing (1.59%). Metabolism emerged as the most prominent category, with subcategories such carbohydrate metabolism (24 annotations) and energy metabolism (17 annotations) being overrepresented. Other metabolic processes included amino acid metabolism (5 annotations), lipid metabolism (3), nucleotide metabolism (2), and metabolism of other amino acids (2) (Figure 3).

#### 2.3.1. Enhanced Metabolism and Cellular Activity at the Globular Stage

In the globular embryos of *J. curcas*, there is an important upregulation of proteins involved in carbohydrate and energy metabolism (Figure 3), particularly enolase (spots 1010 and 955) and L-lactate dehydrogenase (LDH) (spots 1026 and 879). Enolase was found to be over 4-fold upregulated in globular embryos compared to pre-globular embryos (Table 2). This enzyme is a key in glycolysis, catalyzing the conversion of phospho-D-glycerate to phosphoenolpyruvate, a step crucial for starch metabolism. Its coordinated expression with LDH, which converts pyruvate to lactate, suggests a tightly regulated glycolytic process likely aimed at meeting high energy demands during the transition to the globular stage. STRING analysis confirmed predicted co-expression between enolase (JCGZ_26217, JCGZ_26219) and LDH (JCGZ_03382) (Supplementary Figure S2), supporting this interpretation.

This is complemented by the high abundance of tricarboxylic acid (TCA) cycle-associated proteins, such as malate dehydrogenase (spots 1254.2, 568, 1100) along with the presence of ATP synthase proteins (Table 2), indicating an enhanced oxidative metabolism in globular embryos. Such metabolic reprogramming aligns with previous observations in the torpedo-stage SE in *Theobroma cacao* L., where aerobic respiration supports embryo development [34].

#### 2.3.2. Photosynthetic Priming for Autotrophic Growth

A significant upregulation of photosynthesis-related proteins (e.g., oxygen-evolving enhancer proteins 1 and 2, chlorophyll a-b binding protein 8, and chlorophyll a-b binding protein of LHCII) were detected in the globular stage, representing 12% of all differentially abundant proteins (Table 2). The above suggests early activation of photosynthetic activity, potentially in preparation for future autotrophic growth. This finding is similar to globular somatic embryos of *Gossypium hirsutum* (cotton), where photosynthetic proteins were upregulated during SE compared to primary embryogenic callus [31].

#### 2.3.3. Protein Folding and Degradation Mechanisms

The protein folding, sorting, and degradation pathways were also found to be active during the globular stage (Figure 3), as evidenced by the upregulation of proteins involved in folding (protein disulfide isomerase), degradation, such as DSK2a-like protein (spot 402) and proteosome subunit alpha types (spots 1278.1, 1278.2, and 1130.2) (Table 2). Interestingly, the same isoform of proteosome subunit alpha type in spot 1278.1 exhibited low expression in globular versus pre-globular embryos (spot 52.1), possibly indicating post-translational modifications or stage-specific roles that merit further investigation. The ubiquitin–proteasome system regulates various aspects of plant development, including embryogenesis, by removing misfolded or damaged proteins. This activity is especially critical during cellular transitions like the callus-to-embryo and globular-to-torpedo stages, as observed in *Cyclamen* [35].

#### 2.3.4. Translational Regulation: Role of eIF3f

Among the individual proteins detected, two were of particular interest, the eukaryotic translation initiation factor subunit f (eIF3f) and the INDOLE-3-ACETIC ACID CID INDUCIBLE26 (Aux/IAA 26).

eIF3f showed reduced abundance in globular embryos compared to pre-globular embryos. In *A. thaliana*, loss of function of *eIF3f* results in arrest at the globular stage in zygotic embryos, implicating its importance in the normal progression of embryogenesis [36].

eIF3f interacts with the regulatory subunits eIF3e and eIF3h [36]. STRING analysis showed that eIF3f also interacts with proteasome subunits (Figures S2 and S3) supporting a potential interaction between translation control and protein turnover during the pre-globular stage in *J. curcas*.

#### 2.3.5. Auxin Signaling and Embryo Developmental Arrest

**Figure 4:**
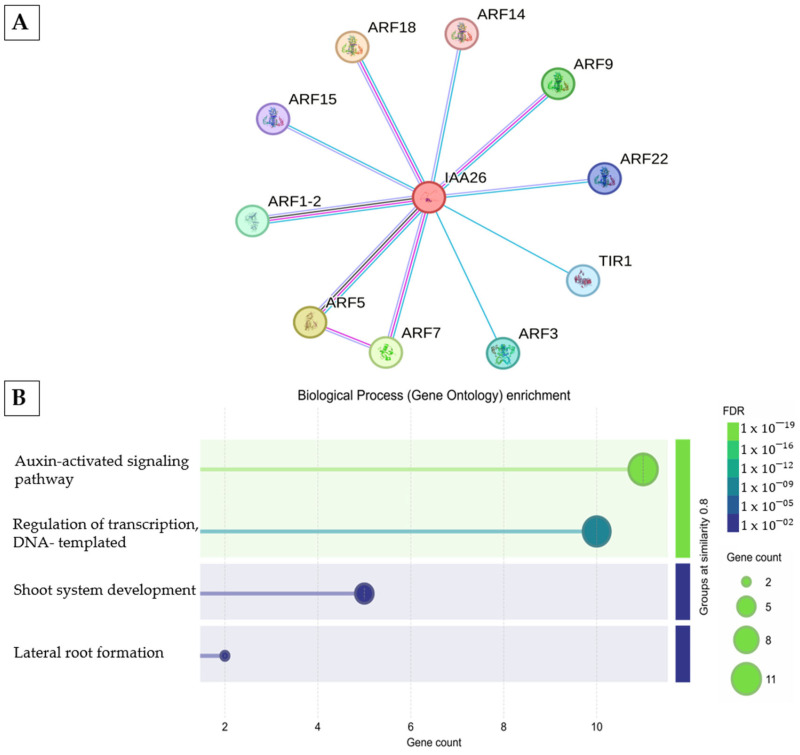
(**A**) Interactome of *J. curcas* Aux/IAA26 protein sequence compared to *A. thaliana* proteins. Colored lines indicate known interactions: a curated database of *A. thaliana* (light blue), experimentally determined (pink), co-expression (black), and homology (violet). (**B**) GO enrichment analysis related to biological processes showed high involvement of Aux/IAA26 in the auxin-activated signaling pathway. The FDR (false discovery rate) measure describes significant enrichment. The protein count in the network is indicated by the dot size. Analysis by STRING database version 12 https://string-db.org/ (accessed on 12 March 2025).

Finally, the protein Aux/IAA26 (a member of the Aux/IAA family) was detected at the globular stage (unique spot 921) (Table S2). Although the protein was not significant in protein abundance (emPAI analysis), other members of the Aux/IAA family are differentially expressed during SE. In *A. thaliana*, mutations in several Aux/IAA genes (*IAA16*, *IAA29*, *IAA30*, and *IAA31*) resulted in a reduced frequency of embryo formation from explants [37]. STRING analysis revealed that Aux/IAA26 interacts with several ARF proteins (ARF1, ARF2, ARF3, ARF5, ARF7, ARF9, ARF14, ARF15, ARF18, ARF22 and TIR1) (Figure 4A), with ARF5 being essential for embryonic axis formation [38]. This interaction network suggests a potential regulatory role for Aux/IAA26 in auxin-mediated signaling pathways (Figure 4B) underlying somatic embryo development and may help explain the developmental arrest observed at the globular stage in *J. curcas* SE.

### 2.4. Gene Expression Analysis in J. curcas

To further elucidate the molecular mechanisms underlying the transition to the globular stage and the observed developmental arrest in *J. curcas* somatic embryos, a gene expression analysis was conducted. This analysis focused on five transcription factors (*LEC1*, *LEC2*, *BBM*, *AGL15*, and *WUS)* and *SERK*. Additionally, the expression levels of eIF3f, a protein with changes between the pre-globular and globular stages in the proteomic analysis, and IAA26, identified in globular embryos, were included in the analysis.

**Figure 5:**
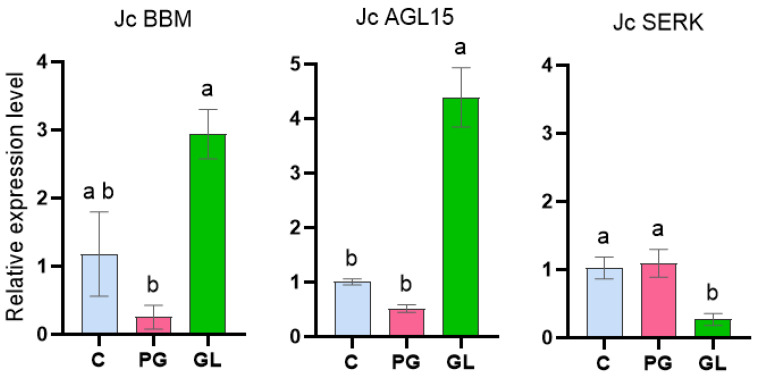
Expression levels of key embryogenic markers: Jc*BBM*, Jc*AGL15*, and Jc*SERK* in callus (C), pre-globular somatic embryos (PG), and globular somatic embryos (GL). The relative transcript levels were normalized to internal control (EF1-α) and calibrated to callus culture. Error bars indicate standard deviation. Different letters above the bars indicate significant differences (*p* < 0.05).

The AP2/ERF transcription factor *BBM* showed an 11-fold increase in expression in globular embryos compared to the pre-globular stage (Figure 5). BBM is considered a master regulator of embryogenic cell competence and identity.

In *A. thaliana*, *BBM* expression is observed in subepidermal cells where somatic embryos initiate [39,40] and is consistently detected in embryogenic cultures but absent from non- embryogenic tissues [41,42]. Similar expression patterns have been reported in other species where *BBM* serves as an excellent marker for embryogenic potential [41,42].

The sharp increase in *BBM* expression during the globular stage in *J. curcas* supports the proteomic and metabolic findings that indicate this stage as a pivotal developmental checkpoint. It also reinforces the idea that although the globular stage is reached, further progression may be hindered, potentially due to misregulation of other essential factors (e.g., eIF3f or Aux/IAA26).

BBM binds to the promoters of several key embryogenesis regulators *LEC1*, *LEC2*, *ABI3*, and *AGL15* during SE in *A. thaliana* [43]. However, in *J. curcas* globular somatic embryos, the expression levels of *LEC1* and *LEC2* were undetectable, likely due to their low expression levels at this stage.

In contrast, *AGL15*, a member of the MADS-box transcription factor family, exhibited an 8-fold increase in expression in globular compared to pre-globular embryos, displaying a gene expression pattern similar to that of *BBM* (Figure 5). This result is similar to previous findings in the same non-toxic *J. curcas* variety from Veracruz, where Jc*AGL15* expression increased 12.7-fold after 42 days in culture during solid SE induction, particularly as embryos approached the globular stage [44]. This strongly supports a conserved role for *AGL15* in promoting SE progression.

Morphological evidence further supports embryogenic identity in these globular structures (Figure 1D–F). When cultures for one month in solid or liquid media supplemented BAP, the globular somatic embryos were capable of forming secondary somatic embryos directly (Supplementary Figure S4). This highlights their developmental competence and reaffirms their status as somatic embryos with the potential for further SE induction.

In contrast, the pre-globular stage showed no significant differential expression of *BBM* and *AGL15* compared to callus. This may be explained by sample heterogeneity as the tissues likely included both pre-globular embryos and non-embryogenic cells. A similar observation was reported in *Coffea arabica*, where low *BBM* expression levels were detected in embryogenic suspensions relative to embryogenic callus. This phenomenon is associated with the fact that the suspensions’ cultures are typically composed of a mixed population, where most of the cells are either quiescent or in the process of degradation and only a portion is embryogenic [41].

AGL15 has been shown to interact with other key transcription factors like BBM and WUS forming part of a regulatory network that promotes SE. However, *WUS* transcripts were not detected in *J. curcas* somatic embryos. In *A. thaliana*, WUS plays a central role in stem cell maintenance in the shoot apical meristem (SAM), and its expression is regulated by auxin gradients during SE induction [45].

Interestingly, AGL15 could be contributing to the developmental arrest observed at the globular stage. AGL15 is known to regulate ethylene metabolism, and ethylene signaling has been suggested to play a significant role during the early stages of embryogenesis, while at a later stage, it disrupts SE development [46]. In *Solanum betaceum*, excessive ethylene accumulation arrested embryo development beyond the globular stage [47]. These findings suggest that high expression or misregulation of *AGL15* may lead to ethylene-induced developmental block in *J. curcas* somatic embryos.

Moreover, AGL15 interacts with SERK through a 14-3-3 protein adaptor [48]. In our study, 14-3-3 protein 6 was upregulated in pre-globular compared to globular embryos (Table 1), suggesting a possible regulatory transition or altered protein–protein interaction dynamics during the globular stage. This observation needs further research to confirm the functional significance of these interactions in *J. curcas* SE.

*SERK* exhibits lower expression levels in globular embryos compared to pre-globular ones (Figure 5). High *SERK*1 expression is associated with cellular reprogramming and acquisition of embryogenic competence, as seen in *Coffea canephora* where *SERK* expression was high in SE at the globular stage [49]. In *A. thaliana*, loss of SERK function disrupts vascular precursor division during the globular stage of zygotic embryo development [50]. This impairment could be linked to the regulation of auxin signaling, including the PIN-FORMED (PIN) proteins, auxin efflux carriers, which are essential for establishing the polar auxin gradients necessary for asymmetric cell division during early embryogenesis in A. *thaliana* [15].

Thus, the low *SERK* expression in globular embryos of *J. curcas* may contribute to the developmental arrest, potentially through the disturbance of auxin gradient establishment and defective asymmetric cell divisions.

Interestingly, when globular somatic embryos were maintained in the liquid SEIM medium for about three months after the third subculture, some embryos began to develop root-like structures (Figure S5). A similar phenotype was observed in *Coffea canephora* when *SERK* expression was transiently silenced, resulting in root-like development, due to the repression of genes encoding auxin-related components and other TFs such as BBM, AGL15, and WUS [49].

**Figure 6:**
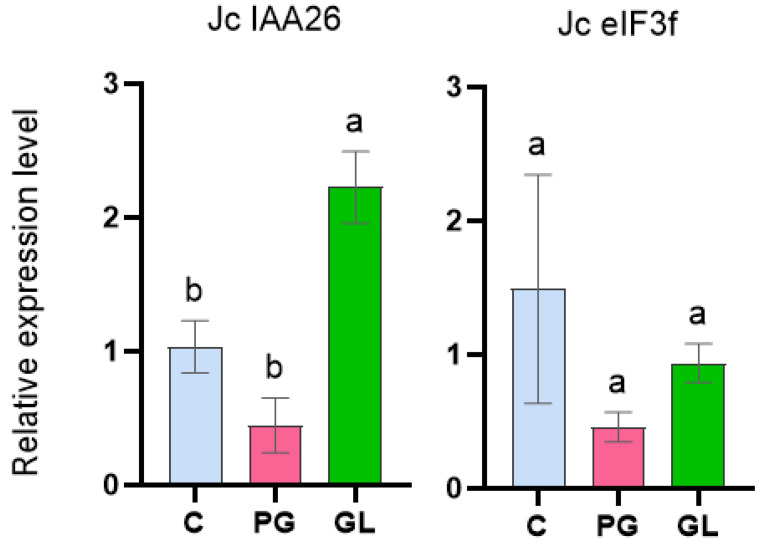
Genes related to plant development obtained from the proteomic study on somatic embryogenesis in *J. curcas*. The graphs display the differential expression levels of the transcription factor Auxin Response Protein *IAA26* (a repressor of early auxin response genes) and the eukaryotic translation initiation factor 3, subunit F (*eIF3f*) in the callus (C), pre-globular somatic embryo (PG), and globular somatic embryo (GL). The relative transcript levels were normalized to the internal control (EF1-α) and calibrated to callus culture. Error bars indicate standard deviation. Different letters above the bars indicate significant differences (*p* < 0.05).

Regarding auxin signaling, the expression analysis of the *IAA26* gene, identified at the proteomic level in globular embryos, was approximately 5-fold higher in globular embryos compared to pre-globular ones (Figure 6). GO enrichment analysis indicated that Aux/IAA26 is significantly involved in the auxin-activated signaling pathway and in the development of the shoot system and lateral root formation (Figure 4B). This suggests an important role played by IAA26 in embryonic patterning and polarity establishment.

STRING analysis revealed that Aux/IAA26 interacts with ARF5 (Figure 4A), which encodes the MONOPTEROS (MP) transcription factor. ARF5/MP is essential for embryonic axis formation in SE *A. thaliana*. Mutations in MP result in severe vascular development defects, further highlighting its importance in early embryogenic patterning [38,51]. These findings suggest that the upregulation of Aux/IAA26 in the *J. curcas* globular embryo may inhibit ARF5 activity, thereby contributing to the developmental arrest observed at this stage.

Regarding the translational machinery, *eIF3f* did not exhibit significant changes at the gene expression level (Figure 6). However, proteomic analysis showed a reduced abundance of eIF3f protein in globular embryos that could still represent a crucial factor contributing to developmental arrest. In *A. thaliana*, the loss of function mutation in *eIF3f* altered the expression of over 3000 genes, many of which are involved in key developmental processes such as pollen tube growth and embryogenesis [36]. Notably, one of the downregulated targets was *AtNAP7*, whose mutation results in embryonic lethality, specifically at the globular stage, showing the critical role of the eiF3F mediated translation in early embryogenic development [36].

In *Arabidopsis thaliana*, several subunits of the eIF3 complex have been found to be regulated by post-translational modifications, such as phosphorylation. eIF3a and eIF3b are modulated in response to light-to-dark transitions, whereas eIF3h is involved in promoting reinitiation of translation [52]. Accordingly, it is possible that eIF3f is similarly regulated at the post-translational level in *J. curcas*, which could influence the synthesis of specific proteins essential for SE. These findings suggest that translational control via eIF3f, in conjunction with auxin signaling modulation by Aux/IAA26, may form part of a developmental checkpoint that determines whether globular embryos proceed toward further differentiation or remain arrested.

## 3. Materials and Methods

### 3.1. Plant Material for Somatic Embryogenesis

Non-toxic seeds of *Jatropha curcas* L. were collected in the field in Veracruz, Mexico, (coordinates 18°26′ N 96°20′ W). The seed coat (the hard layer that covers and protects the surface of the seed) was removed before the disinfection process, which involved a sequential immersion in 70% ethanol for 1 min, 0.6% sodium hypochlorite for 1 min, 70% ethanol for 1 min, 4–6% sodium hypochlorite supplemented with 0.01% Triton X-100 for 3 min, and 0.6% sodium hypochlorite for 7 min. This procedure removed the integument. Seeds were rinsed four times with sterile distilled water. Germination was carried out in 50 mL of developmental medium (DM), consisting of Gamborg’s B5 medium [53] supplemented with 3% (*w*/*v*) sucrose and 25 mg/L cysteine [54]. Cultures were maintained in Magenta™ boxes under controlled environmental conditions (25 ± 2 °C) with a 16 h light/8 h dark photoperiod.

### 3.2. Induction of Callus

Hypocotyls were excised from 14-day-old *J. curcas* seedlings. Two explants were cultured in triplicate flasks with callus induction medium (CIM) consisting of Murashige and Skoog (MS) basal medium [55] supplemented with 0.5 mg/L 2,4-dichlorophenoxyacetic acid (2,4-D) and 2% (*w*/*v*) sucrose. The callus was subcultured on solid maintenance medium (CMM), composed of MS medium with 3% (*w*/*v*) sucrose, 0.01 mg/L 6-benzylaminopurine (BAP), 1 mg/L 1-naphthaleneacetic acid (NAA) and 10 mg/L ascorbic acid. The pH of all media was adjusted to 5.8 prior to the addition of 2 g/L Gelrite and autoclaving. Cultures were incubated at 25 °C under a 16 h light/8 h dark photoperiod. Callus from CMM medium was harvested after two months of culture.

### 3.3. Induction of Somatic Embryos in Liquid Medium

Developing callus cultures were transferred to the liquid somatic embryo induction medium (SEIM), consisting of MS medium supplemented with 3% (*w*/*v*) sucrose, 0.06 mg/L BAP, 0.05 mg/L NAA, and 10 mg/L ascorbic acid. The pH of the medium was adjusted to 5.9 before autoclaving. Cultures were maintained for 8 days, with subcultures performed three times a week in the same medium. Cultures were incubated under continuous light at 126 μmol m^−2^ s^−1^, at 25 °C, and with orbital shaking at 110 rpm. Pre-globular somatic embryos were collected 5 to 7 days after the third subculture, while globular-stage embryos were harvested approximately one month later.

### 3.4. Protein Extraction

Three independent biological extractions were performed for each embryogenic stage (pre-globular and globular), following the protocol reported in Liu et al. [19]. Two grams of the somatic embryos were frozen in liquid nitrogen, pulverized using a mortar and pestle, and equally distributed in two Eppendorf tubes with 1.5 mL extraction buffer each [50 mM Tris-HCl pH 8, 250 mM sucrose, 10 mM EGTA, 1 mM dithiothreitol (DTT), 1% (*v*/*v*) Triton X-100 and 1 tablet of cOmplete^TM^ Mini Protease Inhibitor Cocktail (Roche Diagnostics, Mannheim, Germany) per 10 mL of prepared buffer]. The tubes were vortexed, sonicated for 5 min, incubated on ice for 20 min, and sonicated again for 5 min.

Centrifugation was performed at 3000× *g* for 20 min at 4 °C. The supernatant was collected, and 112.5 μL of trichloroacetic acid was added for every 900 μL of extract. The samples were then incubated for 1 h at 20 °C and centrifuged at 1000× *g* for 5 min; the supernatant was discarded. Protein pellets were washed twice with cold acetone containing 20 mM DTT, and two washed with cold acetone alone. After each wash, centrifugation was performed at 1000× *g* for 5 min at 4 °C. The final pellets were air-dried for 30 min at room temperature and resuspended with 200 μL DeStreak^TM^ Rehydration Solution (GE Healthcare, Chicago, IL, USA) containing optimized concentrations of urea, thiourea, CHAPS, and DeStreak^TM^ Reagent, and 0.5% ampholyte in the pH 4–7 range. After resuspension, samples were centrifuged at 14,000× *g* for 5 min at 4 °C, and the supernatant was collected. Protein concentration was quantified using the 2-D Quant Kit (GE Healthcare, Chicago, IL, USA).

### 3.5. Two-Dimensional IEF/SDS–PAGE and Protein Staining

Three independent electrophoresis runs were performed for each embryogenic stage. In the first gel dimension, proteins were separated according to their isoelectric point, using 200 µg of protein. The homogenized proteins were loaded onto Immobiline ^®^ dry strip gels (11 cm, pH 4–7 non-linear, GE Healthcare, Chicago, IL, USA), and rehydrated at room temperature for 15 h. Isoelectric focusing was conducted using an Ettan IPGphor apparatus (GE Healthcare, Chicago, IL, USA) under the following program: 200 V for 1 h, 500 V for 1 h, 1000 V for 1 h, 6000 V for 2 h and 30 min, and finally for 5400 V/h until completion. All focusing steps were carried out at 20 °C and 50 μA per strip. The IPG strips were equilibrated according to the ReadyPrep^TM^ 2-D Starter Kit (Bio-Rad Laboratories, Hercules, CA, USA).

The second electrophoretic dimension was performed on 12.5% CRITERION^TM^ precast polyacrylamide gels (Bio-Rad Laboratories, Hercules, CA, USA). Separation was conducted at constant voltage of 160 V in a Criterion™ cell of an electrophoretic chamber (Bio-Rad Laboratories, Hercules, CA, USA). SDS-PAGE was performed using a running buffer containing 25 mM Tris-HCl pH 8.8, 192 mM glycine, and 1% SDS. The molecular weight marker (10–250 kDa) Kaleidoscope™ (Bio-Rad Laboratories, Hercules, CA, USA) was used. Following electrophoresis, the gels were fixed in 10% (*v*/*v*) ethanol and 7% (*v*/*v*) acetic acid for 30 min and subsequently stained with Sypro Ruby protein stain (Bio-Rad Laboratories, Hercules, CA, USA) for 20 h. Destaining was performed by incubating the gels in the same fixing solution for 1 h with gentle agitation.

### 3.6. Image Acquisition and Analysis

Three gels for each type of embryogenic stage studied were used for quantitative analysis. Sypro-Ruby-stain gels were scanned and digitized on the ChemiDoc^TM^ MP Imaging System (Bio-Rad Laboratories, Hercules, CA, USA), then were analyzed using ImageMaster^TM^ 2D Platinum software version 7 (Ge Healthcare, Chicago, IL, USA). Protein spots were automatically detected by the software, followed by manual corrections to improve spot recognition and alignment. A one-way ANOVA test based on relative spot volume was applied. Fifty spots with alterations in abundance, with a *p*-value ≤ 0.02, were selected to represent significant differences in protein levels and were analyzed in more detail.

### 3.7. In-Gel Digestion and Mass Spectrometry Analysis

The fifty protein spots of interest were manually excised from the Sypro-Ruby-stain gels, and proteolytic in-gel digestion was conducted with trypsin. Briefly, samples were incubated twice with 200 µL of acetonitrile under gentle agitation for 5 min each. The gel fragments were incubated for 30 min with 50 µL of 10 mM DTT in 100 mM ammonium bicarbonate at 56 °C. The solution was discarded. A measure of 50 µL of 100 mM iodoacetamide in 100 mM ammonium bicarbonate was added for alkylation, followed by incubation for 30 min at room temperature. Then the solution was discarded.

The gel was rehydrated with 50 µL of 100 mM ammonium bicarbonate and acetonitrile through successive incubations for 10 and 5 min, respectively, then dried. In-gel proteolytic digestion was carried out by incubating in 50 µL of trypsin solution (2 ng/µL) for 40 min at 4 °C. Subsequently, 50 µL of 50 mM ammonium bicarbonate was added and it was incubated overnight at 37 °C.

Tryptic peptides were extracted sequentially: first with 5% formic acid and then twice with a mixture of 5% formic acid and 50% acetonitrile. Extracts were stored at −20 °C until sequencing.

Mass spectrometric analysis was performed using the MALDI-TOF/TOF 4800 Plus analyzer (ABSciex, Framingham, MA, USA). Each MS spectrum was acquired by accumulating 1000 shots over a mass range of 850–4000 Da at a laser intensity of 4500. The MS/MS spectra were obtained by fragmenting selected precursor ions using collision-induced dissociation (CID) and were acquired by 3000 shots at a laser intensity of 5000. The generated MS/MS spectra were compared using Protein Pilot software version 2.0.1 (ABSciex, Framingham, MA, USA) against the *Jatropha curcas* (Barbados nut) database (downloaded from UniProt version 2015_6, 27,058 protein sequences) using the Paragon algorithm. Only proteins with an unused score >1.3 (corresponding to 95% confidence) were considered.

Uncharacterized proteins were further analyzed using the NCBI BLAST tool version 2.0.8+ https://blast.ncbi.nlm.nih.gov/Blast.cgi (accessed on 21 June 2018). In cases where more than one protein was found per spot, the exponential protein abundance index [18] was used. The formula applied was as follows:

emPAI = 10^N observed/N observable^ − 1 (1)

where N observed refers to the number of peptides observed experimentally, and N observable refers to the peptides obtained theoretically in in silico digestion.

For this study, N observed includes all peptides that contribute to the unused score and are identified with a 95% confidence level with carbamidomethyl modification and methionine oxidation without cleavages. The N observable was obtained through in silico digestion using the Peptide Mass tool from Expasy https://web.expasy.org/peptide_mass/ (accessed on 14 August 2017), in which digestion with trypsin, monoisotopic mass, and cysteine treated with iodoacetamide as well as methionine oxidation were considered. The mass range was 850 to 4000 Da. Protein content was calculated as follows:

Protein content in % molar = (emPAI/∑(emPAI)) × 100(2)

where ∑(emPAI) represents the sum of all emPAI values from the proteins identified in each spot.

Proteins comprising more than 50% of the total molar protein content per spot were selected. In nine protein spots, this method was insufficient to designate a protein as exhibiting a volume change; therefore, sequences exhibiting a high molar percentage or covering at 95% were selected.

### 3.8. Biological Function

To assign biological functions, GO terms were searched and categorized into GO slim categories of plants using QuickGo https://www.ebi.ac.uk/QuickGO/ (accessed on 22 April 2021). To identify which metabolic pathways are represented in this study, a Kyoto Encyclopedia of Genes and Genomes (KEGG version 5) pathway analysis was performed https://www.genome.jp/kegg/pathway.html (accessed on 8 August 2021), utilizing BLASTKOALA version 2.3 https://www.kegg.jp/blastkoala/ (accessed on 10 August 2021) for genes that had not been annotated. The STRING database version 12 https://string-db.org/ (accessed on 12 March 2025) was used to identify protein–protein interactions.

### 3.9. RNA Extraction and cDNA Synthesis

Total RNA was extracted from callus tissue, pre-globular and globular somatic embryos, following the procedure of Wanqian et al. [56]. Callus and embryo samples were frozen in liquid nitrogen and pulverized using a mortar and pestle. A measure of 250 mg was homogenized in 750 µL extraction buffer (180 mM Tris–HCl pH 8, 4.5 mM EDTA, 90 mM LiCl, 1% SDS, 0.02% (*v*/*v*) ß-mercaptoethanol) and incubated for 10 min at 65 °C. Then, 750 µL of phenol/chloroform/isoamyl alcohol (25:24:1) was added and incubated for 10 min at 25 °C. The upper aqueous phase was collected after centrifugation at 12,400 *g* for 10 min at 4 °C. A measure of 3 M potassium acetate was added to 1/3 of the volume obtained from the aqueous phase, followed by 0.7 volumes of phenol/chloroform/isoamyl alcohol (25:24:1), and the mixture was incubated for 10 min at −20 °C. The samples were centrifuged at 12,400× *g* for 10 min at 4 °C. The upper aqueous phase was collected and cleaned with 0.1 volume of 3 M potassium acetate and 1 volume of cold isopropanol. After the incubation at −20 °C for two hours, the mixture was centrifuged at 12,400× *g* for 10 min at 4 °C. The pellet was washed with 1 mL of cold 70% ethanol, and centrifuged at 7600× *g* for 10 min at 4 °C. Finally, the pellet was dried for 10 min on ice and then homogenized in 20–50 μL of water.

Total RNA was quantified using a Nanodrop 2000 spectrophotometer (Thermo Scientific^TM^, Wilmington, DE, USA), and its integrity was verified through denaturing agarose gel. Contamination with total genomic DNA contamination was eliminated by treating the sample with RNAse-free DNase I (Thermo Scientific^TM^, Vilnius, Lithuania) following the supplier’s instructions.

DNA-free RNA was first prepared following the instructions of the DNase I, RNase-free kit (Thermo Scientific™, Vilnius, Lithuania), with the following modifications: 25 µg of RNA were mixed with 5 µL of 10× reaction buffer (MgCl_2_) and 5 µL of DNase I; the volume was adjusted to 50 µL with sterile water. The mixture was incubated at 37 °C for 30 min. Subsequently, 5 µL of 50 mM EDTA were added, and the samples were incubated at 65 °C for 10 min.

The resulting DNA-free RNA was used as a template for cDNA synthesis using the RevertAid reverse transcriptase (RT) (Thermo Scientific^TM^, Vilnius, Lithuania). In a 200 µL tube, 4 µL of 5× reaction mix, 2 µL of Maxima enzyme mix, 4 µL of RNA, and 10 µL of sterile water were combined. After brief mixing, the samples were incubated sequentially at 25 °C for 10 min, at 65 °C for 30 min, and at 85 °C for 5 min. The cDNA product was stored at −20 °C.

### 3.10. Gene Expression Analysis via RT-qPCR

The expression levels of the genes *BBM*, *LEC1*, *LEC2*, *AGL15*, *WUS*, *SERK*, *eIF3f*, and *IAA26* were analyzed in callus, pre-globular and globular embryos. The primers designed for qPCR are listed in Supplementary Table S3. All qRT-PCR reactions were performed with at least two biological replicates and two technical replicates per sample on a Bio-Rad CFx96 real-time PCR detection system (Bio-Rad^TM^, Hercules, CA, USA) using the Maxima SYBR Green qPCR Master Mix (2X) kit (Thermo Scientific, Waltham, MA, USA). A final reaction volume of 16 µL, containing 1.28 µL of cDNA and 0.1 µM specific primers, was utilized. Thermal cycling conditions were as follows: initial incubation at 50 °C for 2 min, followed by denaturation at 95 °C for 10 min, and 36 amplification cycles consisting of 95 °C for 15 s, 55 °C for 30 s and 72 °C for 30 s. A melting curve was generated by a constant increase in the temperature from 60 to 95 °C in 0.5 °C increments every 5 s; electrophoresis runs were conducted to confirm the formation of a single amplification product. The elongation factor1α (EF1-α) gene was used as an internal control [57], while the callus was used as a reference condition. Gene relative expression was calculated using the 2^−ΔΔCT^ method (ΔΔCT= ΔCT reference condition − ΔCT compared condition).

### 3.11. Statistical Analysis

Statistical analysis was performed using one-way ANOVA (*p* < 0.05), followed by Tukey’s test (*p* < 0.05), with GraphPad Prism version 10.4.1 (GraphPad Software, Boston, MA, USA).

## 4. Conclusions

In this work, we tried to integrate gene expression analysis and proteomic data to delve into the molecular mechanisms that participate in early somatic embryogenesis in *J. curcas*.

The proteomic analysis of pre-globular and globular somatic embryos revealed significant metabolic changes, particularly in pathways related to energy production and protein folding. Some key proteins such as eIF3f and IAA26 showed altered expression patterns, providing potential markers for further investigation into SE regulation in different species, especially those in the Euphorbiaceae family.

The high expression of genes such as *BBM*, *AGL15*, and *IAA26*, along with the reduced expression of *SERK* genes and unchanged expression of *eIF3f* in globular somatic embryos, suggest a possible disruption in auxin distribution. This imbalance may contribute to the developmental arrest observed at the globular stage. Moreover, the discrepancies between transcript levels and protein abundance for eIF3f highlights the relevance of post-translational modifications in controlling embryogenic competence and progression.

These findings provide new insights into early SE regulation in *J. curcas*, offering a platform for future functional studies, and highlights the potential regulatory points that could be manipulated to promote complete embryo development. This study also serves as a foundation for the optimization of SE protocols in *J. curcas*, particularly for the propagation of non-toxic varieties with high biotechnological value.

We are aware that our study presents some limitations as we stopped the analysis in the globular stage and did not see the formation of key structures and cellular differentiation that occurs at later stages. It would be very interesting to incorporate additional studies on different developmental stages, the functional validation of candidate genes, and the optimization of culture conditions to provide a deeper understanding of SE and to overcome the developmental bottleneck, enabling the large-scale production of elite *J. curcas* plants through somatic embryogenesis.

## Figures and Tables

**Table 1:** Functional annotation of selected proteins in pre-globular somatic embryos of *J. curcas*.

ImageMaster^TM^ 2D Platinum Data				QuickGo Data	
Spot Number ^a^	Protein Number ^b^	Fold Change ^c^	Protein Name ^d^	GO SLIM ^e^	GO TERMS ^f^
21	1	4.02	14-3-3 protein 6	__	__
319	1	3.63	Hypothetical protein JCGZ_08406	__	__
558	1	3.23	Endo-1,3;1,4-beta-D-glucanase	__	__
230	3	1.94	Hypothetical protein JCGZ_19572	__	__
215	1	4.33	Fructokinase-2	Carbohydrate metabolic process	Fructose metabolic process; carbohydrate phosphorylation
57	2	4.43	Nucleoside diphosphate kinase	Nucleobase-containing compound metabolic process	GTP, UTP, CTP biosynthetic process
52	1	1.74	Proteasome subunit alpha type	Protein metabolic process	Ubiquitin-dependent protein catabolic process
305	1	5.58	Glutathione S-transferase PARB	Response to chemical stimulus	Glutathione metabolic process; response to toxic substance
57	1	4.43	Superoxide dismutase [Cu-Zn]	Response to stress and chemical stimulus	Superoxide metabolic process; removal of superoxide radicals; cellular oxidant detoxification
239	1	3.64	LysM domain receptor-like kinase 3	Response to stress, biotic stimulus; external stimulus	Innate immune response
404	1	2.92	Eukaryotic translation initiation factor 3 subunit F	Translation	Formation of cytoplasmic translation initiation complex; cytoplasmic translational initiation

The emPAI tool was used alone or in combination with peptide coverage values greater than 95% to select the most abundant proteins in the spots with multiple proteins. ^a^ Number of each spot from 2-DE gel; ^b^ protein number from protein spot from mass spectrometric analysis; ^c^ fold change was calculated as the ratio of the difference between pre-globular relative spot volume versus globular spot volume, relative spot volume (% volume) is defined as the ratio of each spot volume per total spot volume in the gel; ^d^ protein name by UniProt version 2015_6 or NCBI version 2.8.0+; ^e^ GO slim terms from Quick Go analysis; ^f^ common Go terms from Quick Go.

**Table 2:** Functional annotation of selected proteins in globular somatic embryos of *J. curcas*.

ImageMaster^TM^ 2D Platinum Data				QuickGo Data	
Spot Number ^a^	Protein Number ^b^	Fold Change ^c^	Protein Name ^d^	GO SLIM ^e^	GO TERMS ^f^
921	1	unique	PREDICTED: Secoisolariciresinol dehydrogenase-like	__	__
1274	1	unique	Actin-7	__	_
1227	1	unique	LOW QUALITY PROTEIN: receptor-like protein kinase FERONIA	__	_
1278	3	3.6	Secoisolariciresinol dehydrogenase	__	__
629	1	3.47	PREDICTED: Secoisolariciresinol dehydrogenase-like	__	__
362	2	2.07	Plastid-lipid-associated protein, chloroplastic	__	_
575	1	3.43	Cysteine synthase	Biosynthetic process	Cysteine biosynthetic process from serine
1150	1	2.85	Acidic endochitinase	Carbohydrate metabolic process	Carbohydrate metabolic process
307	1	2.55	Sedoheptulose-1,7-bisphosphatase, chloroplastic	Carbohydrate metabolic process	Carbohydrate metabolic process
422	1	3.44	WEB family protein At5g55860 isoform X1	Cellular component organization	Chloroplast avoidance movement; chloroplast accumulation movement
782	1	Unique	Cytochrome b6-f complex iron-sulfur subunit	Cellular process	Transmembrane transport
793	1	6.18	Cytochrome b6-f complex iron-sulfur subunit	Cellular process	Transmembrane transport
266	3	3.7	AtpB	Cellular process	ATP metabolic process
1100	1	Unique	PREDICTED: Malate dehydrogenase	Generation of precursor metabolites and energy	Malate metabolic process
1008	1	Unique	PREDICTED: NADH dehydrogenase	Generation of precursor metabolites and energy	ATP synthesis coupled electron transport
432	1	8.51	ATP synthase subunit beta	Generation of precursor metabolites and energy	ATP biosynthetic process; ATP metabolic process
955	1	5.06	PREDICTED: Enolase-like	Generation of precursor metabolites and energy	Glycolytic process
1010	1	4.59	PREDICTED: Enolase	Generation of precursor metabolites and energy	Glycolytic process
1254	2	4.13	Malate dehydrogenase	Generation of precursor metabolites and energy	Tricarboxylic acid cycle; malate metabolic process
1254	1	4.13	Fructose-bisphosphate aldolase	Generation of precursor metabolites and energy	Glycolytic process
374	1	2.85	Fructose-bisphosphate aldolase	Generation of precursor metabolites and energy	Glycolytic process
175	1	2.18	Probable ribose-5-phosphate isomerase 3, chloroplastic	Generation of precursor metabolites and energy	Pentose-phosphate shunt, non-oxidative branch
440	1	1.73	ATP synthase subunit beta	Generation of precursor metabolites and energy	ATP biosynthetic process; ATP metabolic process
568	1	1.52	PREDICTED: Malate dehydrogenase, chloroplastic	Generation of precursor metabolites and energy	Tricarboxylic acid cycle; malate metabolic process
422	4	3.44	Type I inositol polyphosphate 5-phosphatase 13 isoform X1	Lipid metabolic process	Phosphatidylinositol dephosphorylation
1026	1	Unique	L-lactate dehydrogenase	Metabolic process	Lactate metabolic process; pyruvate metabolic process
879	1	4.57	L-lactate dehydrogenase	Metabolic process	Lactate metabolic process; pyruvate metabolic process
713	2	2.22	3-isopropylmalate dehydratase small subunit 3	Metabolic process	Oxoacid metabolic process
1064	3	3.68	4-hydroxy-4-methyl-2-oxoglutarate aldolase	Nucleic acids metabolic process	Regulation of RNA metabolic process
686	1	13.68	PREDICTED: Chlorophyll a-b binding protein 8, Chloroplastic	Photosynthesis	Photosynthesis, light harvesting
708	1	9.59	PREDICTED: Oxygen-evolving enhancer protein 2, chloroplastic	Photosynthesis	Photosynthesis
704	1	5.42	PREDICTED: Chlorophyll a-b binding protein of LHCII (light harvesting complex II) type 1	Photosynthesis	Photosynthesis, light harvesting
626	1	4.7	PREDICTED: Oxygen-evolving enhancer protein 1, chloroplastic	Photosynthesis	Photosystem II assembly
422	5	3.44	Chlorophyll a-b binding protein 8, chloroplastic	Photosynthesis	Photosynthesis, light harvesting
622	1	2.74	PREDICTED: Oxygen-evolving enhancer protein 1, chloroplastic	Photosynthesis	Photosystem II assembly
402	2	unique	PREDICTED: Ubiquitin domain-containing protein DSK2a-like	Protein metabolic process	Ubiquitin-dependent protein catabolic process
1278	2	3.6	Proteasome subunit alpha type	Protein metabolic process	Ubiquitin-dependent protein catabolic process
1278	1	3.6	Proteasome subunit alpha type	Protein metabolic process	Ubiquitin-dependent protein catabolic process
1130	1	3.11	PREDICTED: Low-temperature-induced cysteine proteinase-like	Protein metabolic process	Proteolysis
1130	2	3.11	Proteasome subunit alpha type	Protein metabolic process	Proteasome-mediated ubiquitin-dependent protein catabolic process
402	1	unique	Protein disulfide-isomerase	Protein metabolic process; response to stress	Protein folding; response to endoplasmic reticulum stress
480	1	4.4	Alcohol dehydrogenase	Response to chemical stimulus	Formaldehyde catabolic process
713	1	2.22	Glutathione peroxidase	Response to stress and chemical stimulus	Response to oxidative stress
1064	1	3.68	Major allergen Pru ar 1	Response to stress, chemical stimulus, endogenous stimulus; signal transduction	Defense response; abscisic acid-activated signaling pathway
816	1	5.44	Major allergen Pru ar 1-like	Response to stress and chemical stimulus, endogenous stimulus; signal transduction	Defense response; abscisic acid-activated signaling pathway; negative regulation of phosphoprotein phosphatase activity

The emPAI tool was used alone or in combination with peptide coverage values greater than 95% to select the most abundant proteins in the spots with multiple proteins. ^a^ Number of each spot from 2-DE gel; ^b^ protein number from protein spot from mass spectrometric analysis; ^c^ fold change was calculated as the ratio of the difference between the relatively globular spot volume versus pre-globular spot volume; ^d^ protein name by UniProt version 2015_6 or NCBI version 2.0.8+; ^e^ GO slim terms from Quick Go analysis; ^f^ common Go terms from Quick Go.

## Data Availability

The data that support the findings of this study are openly available within this manuscript and its supporting materials.

## Supplementary Materials

The following supporting information can be downloaded at: https://www.mdpi.com/article/10.3390/ijms26136384/s1.
