# Supplementary material for: Proteomic Analysis and Expression of Selected Genes During the Early Somatic Embryogenesis of Jatropha curcas L
Source: Int J Mol Sci. 2025 Jul 2;26(13):6384. doi: 10.3390/ijms26136384 (PMC12249898; doi:10.3390/ijms26136384)
Supplement: Supplementary file 1 [file ijms-26-06384-s001.zip › Supplementary Figures Medina-Hernández et al., 2025.pdf]

Supplementary figures

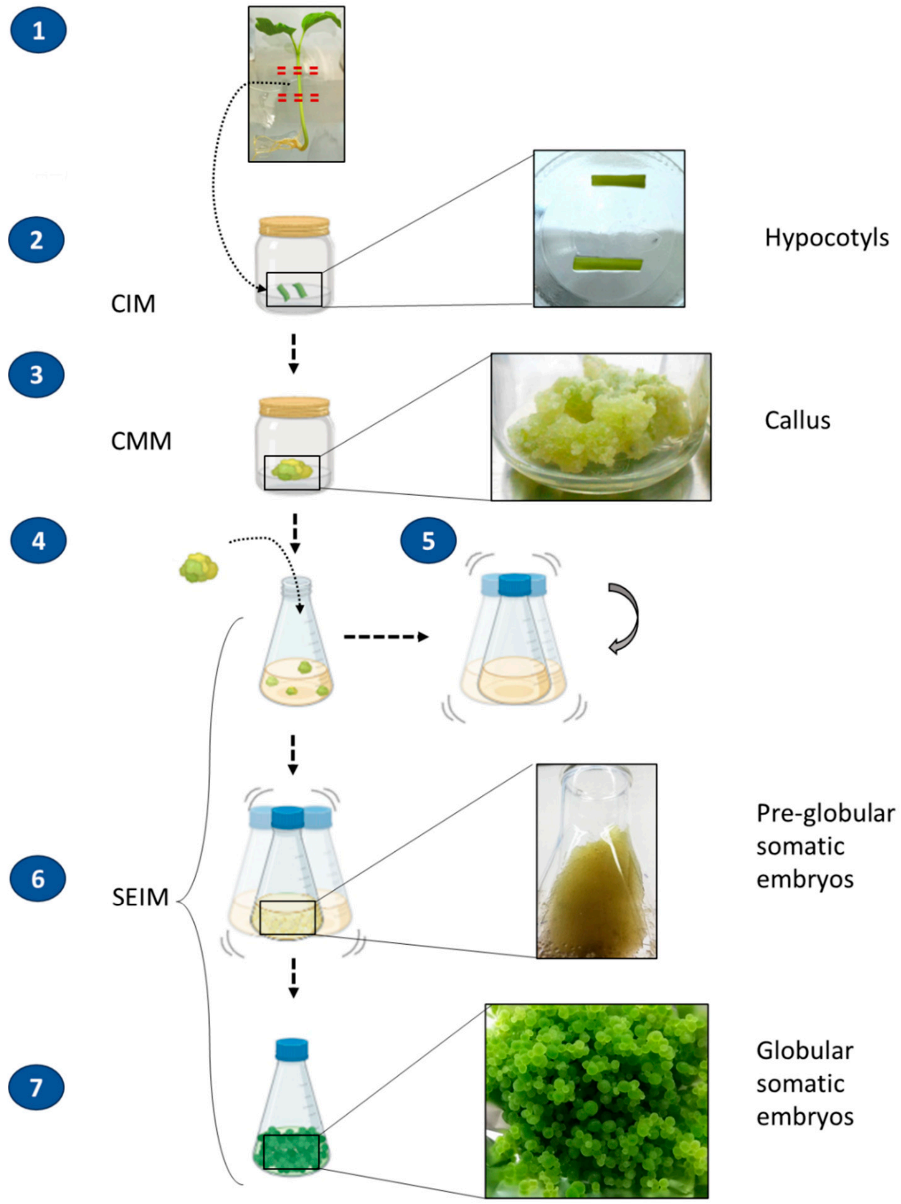

Figure S1. Procedure for the induction of globular somatic embryos of *Jatropha curcas* using liquid medium. 1) Hypocotyl explants were obtained from 14-day-old *J. curcas* seedlings, 2) which were cultivated on callus induction medium (CIM) for one month, 3) induced callus was subculture in callus maintenance medium (CMM) for an additional month when a green friable callus was obtained. 4) Friable callus was transferred to SE induction liquid medium (SEIM) to initiate cell suspension. 5) Suspensions were subculture weekly by decantation to fresh SEIM for three times, 6) creamy yellow pre-globular somatic embryos in suspension were formed after one week of the third subculture, 7) green globular somatic embryos, approximately 0.2 -0.5 cm, were observed a month after the third subculture with weekly medium replacement in the same flask. Created in https://BioRender.com.

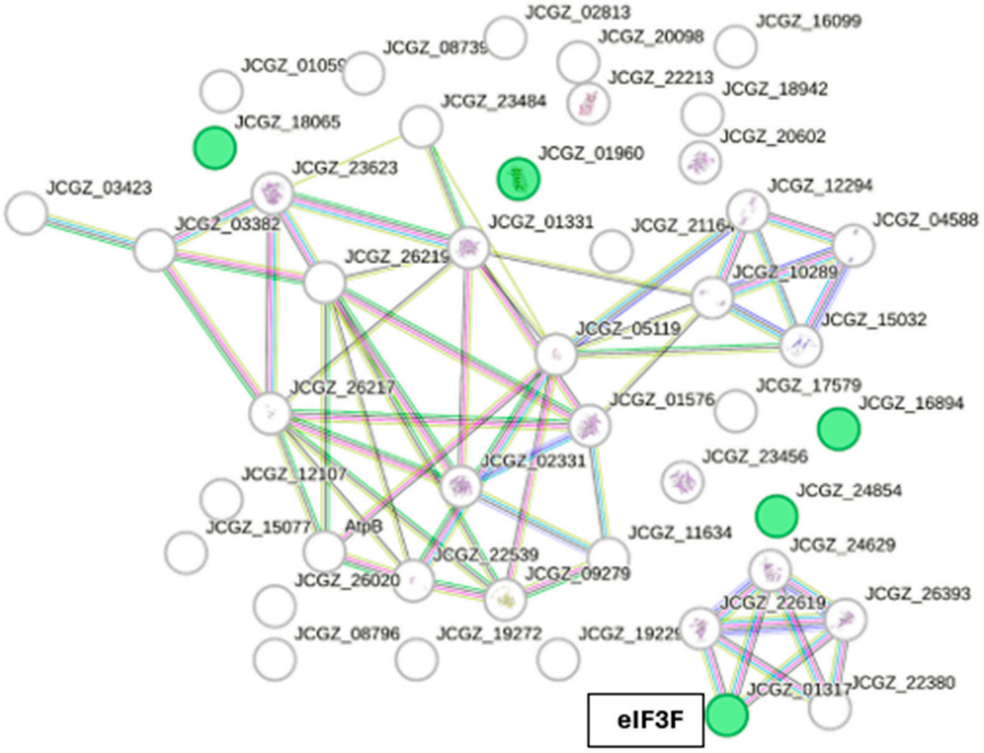

Figure S2. Protein-protein interaction network of proteins upregulated in globular somatic embryos compared to pre-globular embryos in *Jatropha curcas*. The network was generated using STRING database version 12 https://string-db.org/ (accessed on 12 March 2025). The interactome shows significantly more interactions than expected by chance indicating strong functional associations among the proteins. Green nodes indicate proteins encoded by genes that were included in the gene expression analysis. Eukaryotic translation initiation factor 3 subunit F (eIF3f; JCGZ_01317) interacts with proteasome subunits. Colored lines indicated different evidence for the interactions such that light blue lines are for interactions from curated databases of *Jatropha curcas*; experimentally determined interactions (pink); co-expression-based predictions (black) and interactions inferred from homology (violet).

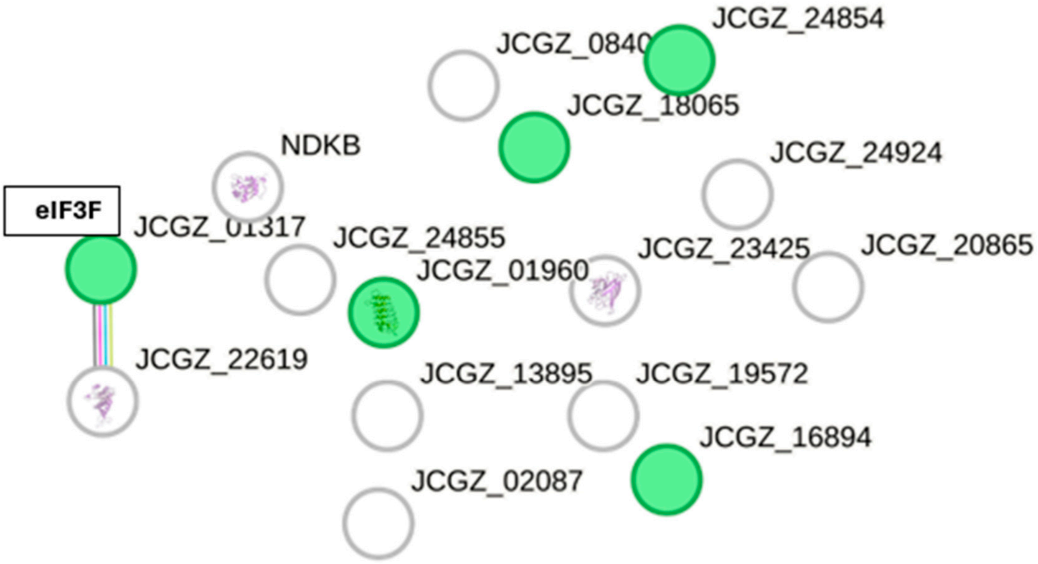

Figure S3. I Protein-protein interaction network of proteins upregulated in pre-globular somatic embryos compared to globular somatic embryos of *Jatropha curcas*. The network was generated STRING database version 12 https://string-db.org/ (accessed on 12 March 2025) and shows no significant enrichment of protein-protein interactions. Only a single interaction was detected between Eukaryotic translation initiation factor 3 subunit F eIF3f (JCGZ_01317) and Proteasome subunit alpha type (JCGZ_22619). Green nodes represent proteins encoded by genes included in gene expression analysis. Colored lines indicated different evidence for the interactions such that light blue lines are for interactions from curated databases of *Jatropha curcas*; experimentally determined interactions (pink); co-expression-based predictions (black) and interactions inferred from homology (violet).

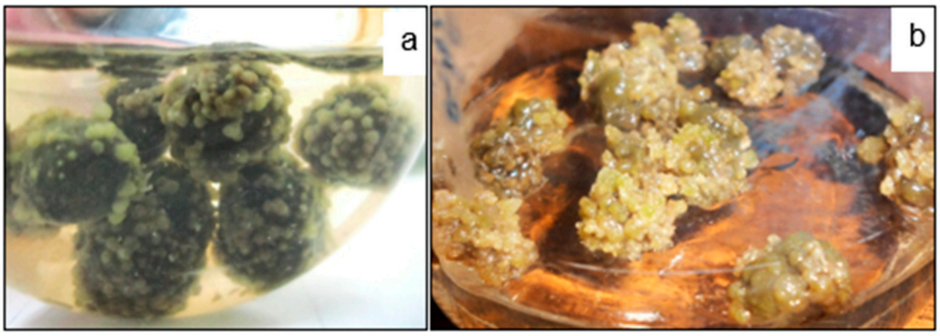

Figure S4. Secondary somatic embryogenesis in *Jatropha curcas*. Globular somatic embryos were cultured in liquid and solid media containing cytokinin leading to the formation of secondary somatic embryos. A) In liquid medium and B) in solid medium, new globular embryos emerged from the surface of primary globular embryos.

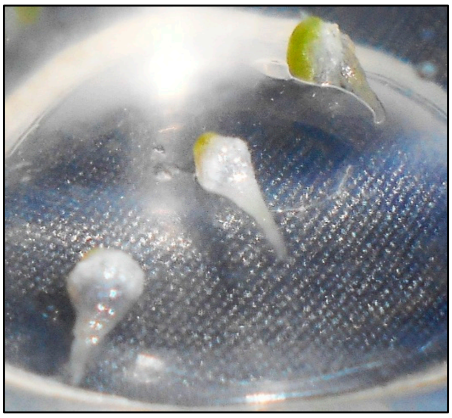

Figure S5. Root formation from *Jatropha curcas* globular somatic embryos. Root-like structures were observed emerging from some globular somatic embryos maintained for more than a month in somatic embryo induction medium (SEIM). The medium was replaced weekly in the same flask, and extended culture conditions appeared to trigger root development in a subset of embryos.
